# A facile hydrothermal approach to the synthesis of nanoscale rare earth hydroxides

**DOI:** 10.1186/s11671-015-0850-2

**Published:** 2015-03-19

**Authors:** Chengyin Li, Hui Liu, Jun Yang

**Affiliations:** State Key Laboratory of Multiphase Complex Systems, Institute of Process Engineering, Chinese Academy of Sciences, Beijing, 100190 China; University of Chinese Academy of Sciences, No. 19A Yuquan Road, Beijing, 100049 China

**Keywords:** Rare earth, Hydrothermal, Hydroxide, Nanorod, Oxide

## Abstract

Nanosized rare earth (RE) hydroxides including La(OH)_3_, Nd(OH)_3_, Pr(OH)_3_, Sm(OH)_3_, Gd(OH)_3_, and Er(OH)_3_ with rod-like morphology are fabricated via a convenient hydrothermal approach. This strategy calls for the first preparation of metal complexes between RE precursors and dodecylamine (DDA) in water/ethanol mixture at room temperature and subsequent thermal decomposition at elevated temperature. The influence of reaction time and water/ethanol volume ratios on the morphology and size of as-prepared RE hydroxides are investigated. CeO_2_ nanoparticles with spherical shape could be directly obtained by hydrothermal treatment of complexes formed between Ce precursors and DDA. In addition, by further calcinating the RE hydroxides at high temperature in air, RE oxide nanorods could be readily produced.

**Electronic supplementary material:**

The online version of this article (doi:10.1186/s11671-015-0850-2) contains supplementary material, which is available to authorized users.

## Background

Recent years have witnessed considerable interest in the design and preparation of rare earth (RE) nanomaterials due to their great potential applications as phosphors, magnets, catalysts, superconductors, and electrolytes [1-8]. In general, the physical and chemical properties of nanomaterials are closely related to their size, chemical composition, and morphology, which render the synthesis of nanosized RE materials an important prerequisite for further scientific or industrial investigations [9]. Among a large number of nanosized RE candidates, the RE hydroxides, which can be easily modified into corresponding oxides, oxysulfides, oxyfluorides, and fluorides, have attracted much attention in recent years [10-14].

So far, the preparation of the nanosized RE hydroxides is mainly based on a hydrothermal/solvothermal treatment in the presence of an inorganic base/organic base at a designed temperature. Typically, a hydrothermal system for preparing RE hydroxides consists of precursor, solvent, and organic additive. The RE precursors are usually simple nitrates or chlorides. The solvent mainly includes water, ethanol, and ethylene glycol. As the physicochemical properties of the solvent can influence reactivity, solubility, and diffusion behavior of the reagents, different solvents benefit morphology and size control. For instance, ethanol, with low RE^3+^ solubility, and ethylene glycol, with high viscosity and tunable diffusion rate of ions, both have been proved to be effective solvents to slow down the nucleation and growth rate of nanoparticles [15]. Besides, a great number of reports have demonstrated that, in the hydrothermal method, the most efficient and straightforward strategy for fine-tuning the shape and size of a targeted material is to select addition of organic additives, including hydrophilic and hydrophobic ones. On the one hand, the coordination effect between the hydrophilic ligands and RE ions will affect the actual concentration of free ions, thereby influencing the concentration of monomer and growth kinetics. On the other hand, the selective adsorption of ligands on different facets of crystallites favors morphology control.

In this work, we demonstrate a hydrothermal approach to the fabrication of RE hydroxide nanorods, labeled as RE(OH)_3_ (RE = La, Nd, Pr, Sm, Gd, and Er). This strategy is based on the thermal decomposition of metal complexes formed by RE precusors and dodecylamine at room temperature. As we will demonstrate, the RE hydroxide nanorods could be further manipulated into corresponding nanosized RE oxides via a simple calcination procedure. Considering the remarkable simplicity of the synthetic approaches, the studies in this work might be promising for creating nanosized RE hydroxides and RE oxides on a large scale for a given technological application (e.g., as phosphors, magnets, and catalysts)

## Methods

### General materials

The RE precursors, including lanthanum(III) nitrate (La(NO_3_)_3_·6H_2_O, 99%), praseodymium(III) nitrate (Pr(NO_3_)_3_·6H_2_O, 99%), neodymium(III) nitrate (Nd(NO_3_)_3_·6H_2_O, 99%), samarium(III) nitrate (Sm(NO_3_)_3_·6H_2_O, 99%), gadolinium(III) nitrate (Gd(NO_3_)_3_·6H_2_O, 99%), and erbium(III) nitrate (Er(NO_3_)_3_·5H_2_O, 99.9%), were from Aladdin Reagents, Shanghai, China; cerium(III) nitrate (Ce(NO_3_)_3_·6H_2_O, 99%) was from Sinopharm Chemical Reagent Co., Ltd., Beijing, China; ethanol (99.5%) was from Beijing Chemical Works, Beijing, China; and dodecylamine (DDA, 98%) was from J&K Scientific Ltd., Beijing, China. All glassware and autoclave Teflon liner were cleaned with *aqua regia*, followed by copious rinsing with deionized water before drying in an oven.

### Synthesis of lanthanide hydroxide nanoparticles

In a typical synthesis of RE hydroxide nanorods, 0.2 mmol of RE precursors (La(NO_3_)_3_, Pr(NO_3_)_3_, Nd(NO_3_)_3_, Sm(NO_3_)_3_, Gd(NO_3_)_3_, Er(NO_3_)_3_, or Ce(NO_3_)_3_) was dissolved in 10 mL of deionized water, and then 10 mL of ethanol containing 5 mL of DDA was added. After sufficient mixing, the mixture was transferred into an autoclave with a volume of 50 mL, which was kept at 180°C for 18 h. After the hydrothermal process, the autoclave was cooled down to room temperature naturally, and the precipitates were collected by centrifugation and washed with deionized water and pure ethanol for several times. Finally, the products were dried at 60°C for structural characterizations. Further, the RE oxides (La_2_O_3_, Pr_6_O_11_, Nd_2_O_3_, and Er_2_O_3_) were produced by calcinating their corresponding RE hydroxides at 600°C for 2 h in air.

### Characterization

Fourier transform infrared spectroscopy (FTIR) analysis was performed on a Bruker Alpha RT-DLATGS spectrometer (Bruker Optik Gmbh, Ettlingen, Germany). The spectra were collected over the range of 400 to 4,000 cm^−1^ in the transmission mode. Powder X-ray diffraction (XRD) measurements were carried out on a Bruker D8 focus X-ray diffractometer (Bruker Optik Gmbh, Ettlingen, Germany), using Cu-Kα radiation (*λ* = 1.5406 Å). TEM and high-resolution TEM (HRTEM) were performed on the JEOL JEM-2100 (JEOL Ltd., Akishima, Tokyo, Japan) and FEI Tecnai G^2^ F20 electron microscope (FEI, Hillsboro, OR, USA) operating at 200 kV with a supplied software for automated electron tomography. For the TEM measurements, a drop of the nanoparticle solution was dispensed onto a 3-mm carbon-coated copper grid. Excessive solution was removed by an absorbent paper, and the sample was dried at room temperature in air.

## Results and discussion

It has been demonstrated that the DDA can interact with almost all transition and noble metal ions to form metal complexes soluble in non-polar organic solvents [16]. Similarly, upon mixing the aqueous solution of RE precursors and ethanolic solution of DDA, metal complexes composed of RE precursors and DDA could also be formed. Ethanol was used to ensure the sufficient contact between RE precursors and DDA since it is water-miscible and a good solvent for DDA. The formation of RE precursor-DDA complexes could be verified by the FTIR spectra of the compounds recovered from the mixture of aqueous RE precursor solution and ethanolic DDA solution. As shown in Additional file 1: Figure S1 for the FTIR spectra of pure DDA, the bands at 3,338, 2,918, and 1,473 cm^−1^ are attributed to the stretching vibrations of C-N, C-H, and N-H, respectively. In comparison with that of pure DDA, apparent differences could be identified in the N-H and C-N stretching vibrations while other FTIR characteristic peaks are remained in RE precursor-DDA compounds (Additional file 1: Figure S1), demonstrating that there was a strong coordination effect between the NH_2_ group in DDA and RE precursors.

**Figure 1:**
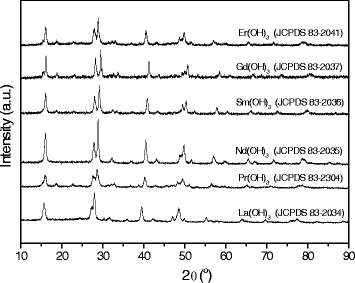
**XRD patterns of RE hydroxides as prepared by decomposing the RE precursor-DDA complexes at elevated temperature.**

The RE precursor-DDA complexes are then decomposed at elevated temperature, resulting in the generation of RE hydroxide products, labeled as RE(OH)_3_, which subsequently grow into RE(OH)_3_ nanorods and are protected by DDA. The possible mechanism accounting for the formation of RE(OH)_3_ might resemble the preparation of lanthanide hydroxide using oleylamine [13] or triethylamine [17] as organic additives, the protonation of which leads to the generation of hydroxyl ions from water solvent for the formation of hydroxides. In this work, DDA provides the basic environment for the formation of RE hydroxides. Figure 1 shows the XRD patterns of RE(OH)_3_ samples. All the diffraction peaks can be readily indexed to the pure hexagonal phase. The peaks are steep and high, and no additional peaks of other phases have been found, indicating that the RE(OH)_3_ products are well crystallized and of high purity. The lattice constants (a and c) were calculated and listed in Additional file 1: Table S1. The decrease in lattice constants from Pr to Er hydroxides can be attributed to the well-known lanthanide contraction [18,19].

**Figure 2:**
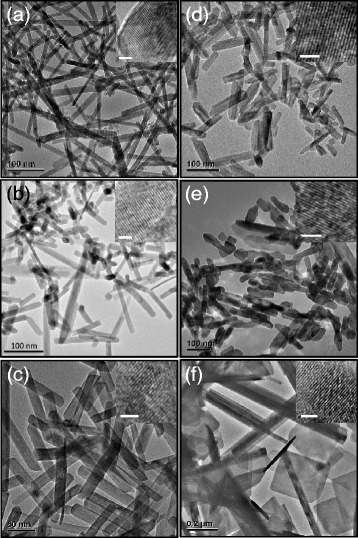
**TEM and HRTEM images.** La(OH)_3_ **(a)**, Pr(OH)_3_ **(b)**, Nd(OH)_3_ **(c)**, Sm(OH)_3_ **(d)**, Gd(OH)_3_ **(e)**, and Er(OH)_3_ **(f)** as synthesized by decomposing the RE precursor-DDA complexes at elevated temperature. The scale bar for the HRTEM images (inset of each TEM image) is 2 nm.

The size and morphology of the RE hydroxide products were examined by TEM. As exhibited in Figure 2, most of these hydroxide products have rod-like structure with lengths up to a few hundreds of nanometers. The histograms resulting from counting 100 well-separated particles (Additional file 1: Figure S2) depict that the average diameters are of ca. 10 to 50 nm (12.2 nm for La(OH)_3_, 14.2 nm for Pr(OH)_3_, 18.8 nm for Nd(OH)_3_, 33.4 nm for Sm(OH)_3_, 18.3 nm for Gd(OH)_3_, and 44.2 nm for Er(OH)_3_, respectively). In addition, for Er(OH)_3_, a number of nanosheets are also observed in the TEM image (Figure 2f). The HRTEM images (insets of each TEM image) indicate that the RE hydroxide nanorods are highly crystallized. It has been believed that the driving force for the anisotropic growth of RE(OH)_3_ nanorods derives from the inherent crystal structure of RE(OH)_3_ materials. As indicated by the XRD patterns (Figure 1), the as-prepared RE hydroxides have hexagonal structures. Their *P*6_3_/*m* space group is usually favorable to the formation of hexagonal rods or plates [9]. However, the influence of the DDA on the anisotropic growth of RE(OH)_3_ cannot be ruled out. The anisotropic adsorption of DDA on the surface of the growing RE(OH)_3_ products and the structure of RE precursor-DDA complexes might have contribution to the formation of RE(OH)_3_ nanorods, analogous to the worm-like Pd nanoparticles derived from coordinating compounds formed between PdCl_2_ and DDA [16].

A special case was observed for the cerium system. Instead of Ce(OH)_3_ nanorods, the hydrothermal treatment of complexes formed by CeNO_3_ and DDA at 180°C results in the formation of CeO_2_ nanoparticles with spherical shapes, as shown by the TEM and HRTEM images in Additional file 1: Figure S3. The nanoparticles are well defined and have an average diameter of ca. 13.2 nm. The XRD pattern (Additional file 1: Figure S3b) illustrates that the CeO_2_ nanoparticles prepared this way have cubic structure (JCPDS card file: 780694). It should be emphasized that although we cannot rule out the Ce(OH)_3_ which is an intermediate product during the hydrothermal treatment of Ce(NO_3_)_3_-DDA complexes, all attempts (e.g., altering temperature, DDA/Ce(NO_3_)_3_ ratios, reaction time, or water/ethanol volume ratios) to prepare Ce(OH)_3_ products were unsuccessful. This may be interpreted that Ce(OH)_3_ is quite unstable and easy to be decomposed at elevated temperature. The Ce(III) ions are then further oxidized to Ce(IV) by air, leading to the generation of CeO_2_ nanoparticles protected by DDA. The stabilization by capping agent (DDA) is important to retain the crystal structure of CeO_2_ as the cubic phase is not a common product under ambient conditions for light and medium RE oxides [9].

After hydrothermal treatment, the FTIR spectra of the products (Additional file 1: Figure S4) provides evidence for the presence of hydroxyl groups, as indicated by the strong bonds around 3600 cm^−1^ (except for Ce(III)-DDA system). In addition, after copious washing by water and ethanol, the DDA, which serves as capping agent for the hydroxide or oxide products, is still detectable in the FTIR spectra, as displayed by the stretching vibrations of N-H (ca. 1,500 cm^−1^) and C-H (ca. 2,900 cm^−1^).

**Figure 3:**
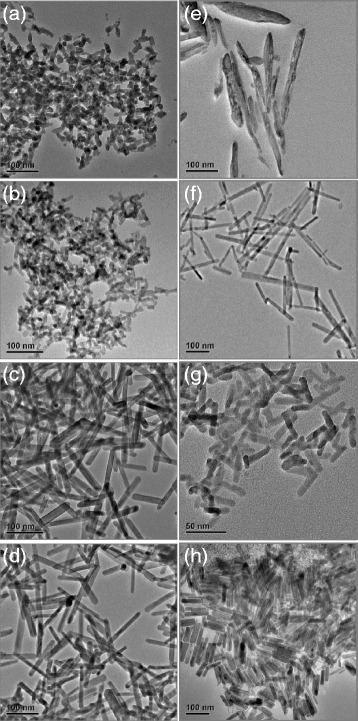
**TEM images of La(OH)**_**3**_ **nanorods.** Prepared under different reaction times and water/ethanol volume ratios: **(a-d)** 6, 12, 18, and 24 h while DDA volume and water/ethanol volume ratio are fixed at 5 mL and 1/1, respectively; **(e-h)** water/ethanol volume ratio of 100/0, 80/20, 20/80, and 0/100 while DDA and reaction time are fixed at 5 mL and 18 h, respectively.

Taking La(OH)_3_ as a typical example, we have conducted a series of experiments under different hydrothermal conditions to investigate the growth of RE hydroxide nanorods. We found that the reaction time and water/ethanol volume ratio have significant influence on the size and morphology of the RE hydroxides, while the effect from the hydrothermal temperature and DDA/RE precursor ratio is only slight. Figure 3a,b,c,d shows the TEM images of La(OH)_3_ nanorods as prepared by hydrothermal treatment of La(NO_3_)_3_-DDA complexes at 180°C for 6, 12, 18, and 24 h, respectively. At 6 h, only short La(OH)_3_ nanorods are observed under TEM (Figure 3a). At longer time (12 h), the length of the nanorods become larger while the diameter of the rods are remained (Figure 3b), indicating that the anisotropic growth of La(OH)_3_ occurs along the axial direction. After 18 h, the La(OH)_3_ nanorods are fully developed, and further increase in reaction time would not alter the morphology and size of the final La(OH)_3_ nanorods (Figure 3c,d).

The TEM images of the La(OH)_3_ nanorods as prepared by the hydrothermal approach under different water/ethanol volume ratios are shown in Figure 3e,f,g,h. As indicated, at water/ethanol volume ratios of 100:0 (Figure 3e), 80:20 (Figure 3f), 20:80 (Figure 3g), and 0:100 (Figure 3h), La(OH)_3_ nanomaterials with rod-like morphology are formed as dominant products. However, as illustrated by these TEM images, the La(OH)_3_ nanorods synthesized at water/ethanol ratio of 80:20 have a larger aspect ratio (Figure 3f), while those synthesized at water/ethanol ratio of 0:100 are more uniform in length and diameter (Figure 3h).

It is well known in colloidal chemistry method that nanoparticles are formed via two processes, i.e., nucleation and crystal growth [20,21]. The nucleation is usually determined by the supersaturation and temperature, while the particle growth is a process of the assembling of atoms on the surface of nuclei, which could be controlled by the diffusion of atom to the growing surface followed by the incorporation into the lattice. The incorporation process might be associated with the formation of chemical bond, which could be regarded as a reaction step. Both the diffusion step and the reaction step can affect the particle formation. The compromise of diffusion step and reaction step might be an important prerequisite to facilitate the formation of nanoparticles with uniform size and narrow size distribution [22]. The ratio of water/ethanol could affect the diffusion of La(OH)_3_ reactants generated from the decomposition of La(NO_3_)_3_-DDA complexes by effectively regulating the viscosity of the mixture and hence results in the formation of La(OH)_3_ nanorods with different sizes and morphologies.

**Figure 4:**
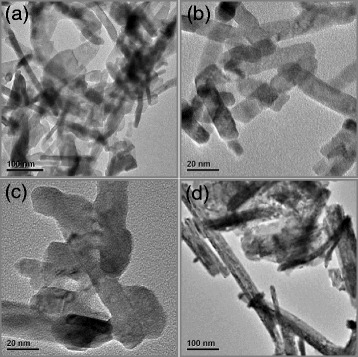
**TEM images of La**_**2**_ **O**_**3**_ **(a), Pr**_**6**_ **O**_**11**_ **(b), Nd**_**2**_ **O**_**3**_ **(c), and Er**_**2**_ **O**_**3**_ **(d) nanorods.** Synthesized by calcinating their corresponding hydroxides at 600°C for 2 h.

By further calcinating the RE(OH)_3_ hydroxides at 600°C for 2 h, corresponding RE oxides could be obtained easily [17,23,24]. La_2_O_3_, Pr_6_O_11_, Nd_2_O_3_, and Er_2_O_3_ have been prepared this way and confirmed by XRD patterns (Additional file 1: Figure S5), which manifest the as-obtained La_2_O_3_ and Nd_2_O_3_ oxides that remain the hexagonal structure of their corresponding hydroxides, while the Pr_6_O_11_ and Er_2_O_3_ are converted into cubic phase after calcination. TEM was used to examine the morphology and size of the as-prepared RE oxides. As shown in Figure 4, in comparison with that of corresponding RE hydroxides, although the rod-like morphology is briefly maintained, significant changes in length, diameter, and dispersivity are indeed noted. Optimal calcination conditions to produce RE oxides with well-defined morphology and uniform diameter are yet to be studied in detail.

## Conclusions

In summary, we have successfully synthesized the RE (RE = La, Nd, Pr, Sm, Gd, and Er) hydroxide nanorods via a convenient hydrothermal synthetic route. This strategy is called for the first preparation of metal complexes between RE precursors and DDA in water/ethanol mixture at room temperature and subsequent thermal decomposition at elevated temperature. Instead of Ce(OH)_3_ nanorods, this hydrothermal approach directly resulted in the formation of spherical CeO_2_ nanoparticles with very uniform size distributions. By further calcinating the corresponding RE hydroxides at high temperature in air, nanosized RE oxides with remained rod-like morphology could be readily obtained. It is expected that the synthetic route developed in this work could be extended to generate other RE-based nanomaterials.

## Additional file

Additional file 1: **Supplementary material.** **Figure S1.** FTIR spectra of pure DDA and RE precursor-DDA complexes recovered from the mixture of aqueous RE precursor solution and ethanolic DDA solution. **Table S1.** Textural property of RE(OH)_3_. **Figure S2.** Histogram of La(OH)_3_ (a), Pr(OH)_3_ (b), Nd(OH)_3_ (c), Sm(OH)_3_ (d), Gd(OH)_3_ (e), and Er(OH)_3_ (f) as synthesized by decomposing the RE precursor-DDA complexes at elevated temperature. The average diameter and related standard derivation are labeled in corresponding figures. **Figure S3.** TEM image (a), HRTEM image (inset of a), and XRD pattern of CeO_2_ nanoparticles as prepared by hydrothermal treatment of complexes formed between CeNO_3_ and DDA. The scale bar for the inset HRTEM image is 2 nm. Figure S4: FTIR spectra of pure DDA and RE precursor-DDA complexes after hydrothermal treatment at elevated temperature.
