# Supplementary material for: A facile hydrothermal approach to the synthesis of nanoscale rare earth hydroxides
Source: Nanoscale Res Lett. 2015 Mar 19;10:144. doi: 10.1186/s11671-015-0850-2 (PMC4385291; doi:10.1186/s11671-015-0850-2)
Supplement: Supplementary file 1 — Supplementary material. Figure S1. FTIR spectra of pure DDA and RE precursor-DDA complexes recovered from the mixture of aqueous RE precursor solution and ethanolic DDA solution. Table S1. Textural property of RE(OH)3. Figure S2. Histogram of La(OH)3 (a), Pr(OH)3 (b), Nd(OH)3 (c), Sm(OH)3 (d), Gd(OH)3 (e), and Er(OH)3 (f) as synthesized by decomposing the RE precursor-DDA complexes at elevated temperature. The average diameter and related standard derivation are labeled in corresponding figures. Figure S3. TEM image (a), HRTEM image (inset of a), and XRD pattern of CeO2 nanoparticles as prepared by hydrothermal treatment of complexes formed between CeNO3 and DDA. The scale bar for the inset HRTEM image is 2 nm. Figure S4: FTIR spectra of pure DDA and RE precursor-DDA complexes after hydrothermal treatment at elevated temperature. [file 11671_2015_850_MOESM1_ESM.docx]

Supplementary Material

**A facile hydrothermal approach to the synthesis of nanoscale rare earth hydroxides**

Chengyin Li,^1,2^ Hui Liu,^1^ and Jun Yang^1,^*

^1^ State Key Laboratory of Multiphase Complex Systems, Institute of Process Engineering, Chinese Academy of Sciences, Beijing 100190, China. Fax: 86-10-8254 4915; Tel: 86-10-8254 4915; E-mail: [jyang@ipe.ac.cn](mailto:jyang@mail.ipe.ac.cn)

^2^ University of Chinese Academy of Sciences, No. 19A Yuquan Road, Beijing 100049, China

Financial support from the 100 Talents Program of the Chinese Academy of Sciences, and National Natural Science Foundation of China (No.: 21173226, 21376247) is gratefully acknowledged.

**Figure S1** FTIR spectra of pure DDA and RE precursor-DDA complexes recovered from the mixture of aqueous RE precursor solution and ethanolic DDA solution.

**Table S1** Textural property of RE(OH)_3_

| RE | RE(OH)_3_ lattice constants (Å) | |
| --- | --- | --- |
|  | a | c |
| La | 6.547 | 3.854 |
| Pr | 6.456 | 3.769 |
| Nd | 6.418 | 3.743 |
| Sm | 6.368 | 3.683 |
| Gd | 6.329 | 3.631 |
| Er | 6.243 | 3.527 |

**Figure S2.** Histogram of La(OH)_3_ (a) , Pr(OH)_3_ (b), Nd(OH)_3_ (c), Sm(OH)_3_ (d), Gd(OH)_3_ (e), and Er(OH)_3_ (f) as-synthesized by decomposing the RE precursor-DDA complexes at elevated temperature. The average diameter and related standard derivation are labeled in corresponding figures.

**Figure S3** TEM image (a), HRTEM image (inset of a), and XRD pattern of CeO_2_ nanoparticles as-prepared by hydrothermal treatment of complexes formed between CeNO_3_ and DDA. The scale bar for the inset HRTEM image is 2 nm.

**Figure S4** FTIR spectra of pure DDA and RE precursor-DDA complexes after hydrothermal treatment at elevated temperature.

**Figure S5** XRD patterns of RE oxides as-prepared by calcinating corresponding RE hydroxides at 600°C for 2 h.
